# Support Vector Machine Regression for Calibration Transfer between Electronic Noses Dedicated to Air Pollution Monitoring

**DOI:** 10.3390/s18113716

**Published:** 2018-11-01

**Authors:** Rachid Laref, Etienne Losson, Alexandre Sava, Maryam Siadat

**Affiliations:** Laboratoire de Conception, Optimisation et Modélisation des Systèmes, LCOMS EA 7306, Université de Lorraine, 57000 Metz, France; rachid.laref@univ-lorraine.fr (R.L.); etienne.losson@univ-lorraine.fr (E.L.); alexandre.sava@univ-lorraine.fr (A.S.)

**Keywords:** calibration transfer, direct standardization, support vector machine regression, electronic nose, air pollution monitoring, gas sensors

## Abstract

Recently, the emergence of low-cost sensors have allowed electronic noses to be considered for densifying the actual air pollution monitoring networks in urban areas. Electronic noses are affected by changes in environmental conditions and sensor drifts over time. Therefore, they need to be calibrated periodically and also individually because the characteristics of identical sensors are slightly different. For these reasons, the calibration process has become very expensive and time consuming. To cope with these drawbacks, calibration transfer between systems constitutes a satisfactory alternative. Among them, direct standardization shows good efficiency for calibration transfer. In this paper, we propose to improve this method by using kernel SPXY (sample set partitioning based on joint x-y distances) for data selection and support vector machine regression to match between electronic noses. The calibration transfer approach introduced in this paper was tested using two identical electronic noses dedicated to monitoring nitrogen dioxide. Experimental results show that our method gave the highest efficiency compared to classical direct standardization.

## 1. Introduction

Nowadays, governmental authorities monitor air pollutants by using a limited number of measurement stations because of the high cost of these instruments, their maintenance, and their cumbersome nature [1]. Within the low spatial resolution given by these stations of air quality monitoring, the data are combined with some modeling software in order to estimate the average pollution in urban areas. However, the dispersion of pollution is a very complex phenomenon that is highly affected by environmental factors and the urban structure. Therefore, densifying the network of air pollution monitoring to get a high spatial and also temporal resolution is a real necessity [2,3].

Due to technological advances, low cost sensors are now able to measure very low pollutant gas concentrations (parts per billion, ppb), which make them promising solutions to cope with the low resolution of the currently implemented air pollution monitoring networks [4,5,6]. During recent years, many studies have revealed that electrochemical and metal oxide gas sensors (MOX) can perform at a ppb level of concentration [7]. Metal oxide gas sensors tend to have higher sensitivity but suffer from the lack of reproducibility and stability. Electrochemical sensors present better stability and selectivity but have a short lifetime [8]. The major difficulties of low cost gas sensors as reviewed in the literature can be summarized in two aspects [9]:

1. Those related to the working principle of the sensors such as the dynamic boundaries, systematic errors, non-linear responses and signal drifts.
2. Those caused by external error sources, such as environmental dependencies and low selectivity.

To overcome these limitations, one can use a system called an electronic nose made up of a sensors array associated with machine learning algorithms [10]. Using an electronic nose allows the exploitation of all the information from its sensors, including the cross-sensitivity and interference from environmental factors, to create a calibration model able to successfully predict pollutant concentrations [11]. After the establishment of this initial calibration model, the other issues limiting the use of electronic noses are related to sensor response changes after a certain period of time or after sensor replacement. Consequently, the current calibration model becomes invalid and a new one must be generated [12]. Each of the units should also be calibrated individually due to poor reproducibility in the sensors fabrication process [13].

Techniques such as calibration transfer are an attractive solution to extend the lifetime of the calibration model by allowing the use of one calibration model for different units. Calibration transfer first appeared in the spectroscopy domain and has since been extended to the gas sensors array [14]. Before presenting transfer calibration techniques, we consider that the transfer calibration is performed between two units, respectively called the master unit and the slave unit. The slave unit may be a different unit identical to the master unit, or it may be the master unit itself over time. Calibration transfer techniques can be divided into two categories. The first one is based on removing the dissimilarity between the master and the slave units by transforming sensor responses of both units to become more similar before creating a calibration model. Among the methods that use this technique, we can cite Orthogonal Signal Correction (OSC) [15], Component Correction (CC) [16] and Generalized Least Squares Weighting (GLSW) [17]. The second category attempts to modify the data of the slave unit to be similar to that of the master unit. These techniques allow the use of the calibration model of the master unit for the data of the slave unit. This family of calibration transfer, called standardization methods, can be classified into three classes according to their strategies [18]:

- Standardization of the model coefficients: consists of modifying the calibration model, which is built on the master unit so as to be suitable for data from the slave unit.
- Standardization of the sensors’ responses: based on transforming the sensors’ responses of the slave unit to be similar to those of the master unit. The calibration model of the master unit then becomes usable for the slave unit.
- Standardization of the predicted values: the predictive values from the slave unit are corrected using a linear relationship calculated between predictive values of the master and the slave units. All standardization methods need a set of known samples from both units (slave and master) that allows matching between them.

The choice among standardization methods depends on the application itself, whether it is a simple or complex system [18]. However, standardization of sensors response methods such as direct standardization (DS) [19] and piece wise direct standardization (PDS) [20] are the most popular.

In this work, we focused on DS and we proposed to use the kernel SPXY (sample set partitioning based on joint x-y distances) algorithm for selecting standardization samples. We also utilized the support vector machine regression (SVM) instead of the classical transformation matrix to make the calibration transfer. For this purpose, we considered the case of two identical systems exposed to the same gas concentrations under the same conditions. The rest of this paper is organized as follows: in Section 2 we describe the experiment setup and data collection process performed in the laboratory. In Section 3, we introduce the methods and algorithms used to make the calibration transfer. We compared the performance of our method with the performance of existing classical direct standardization method in Section 4. Finally, a conclusion of this work and further directions are given in Section 5.

## 2. Experiment Setup and Data Collection

**Figure 1:**
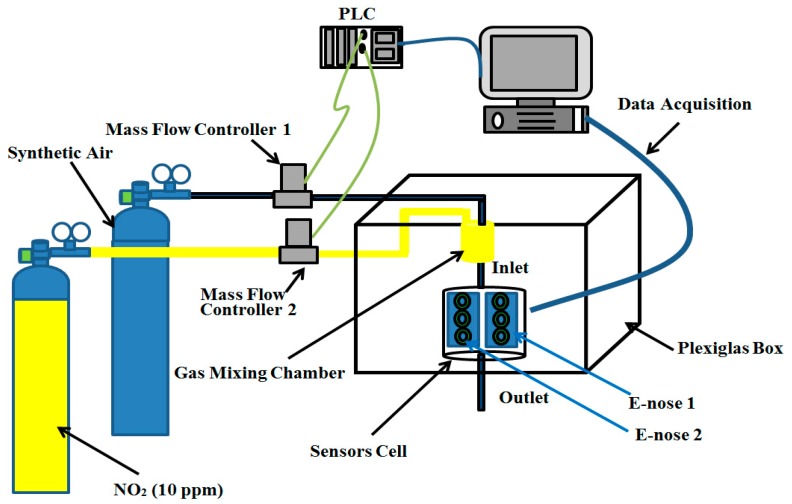
Schematic representation of the experimental setup. NO_2_: nitrogen dioxide.

We have designed two identical E-noses for nitrogen dioxide (NO_2_) monitoring. Each one was made up of three gas sensors, an electrochemical sensor (NO2-B43F from Alphasense, Essex, UK), and two MOX sensors (MICS-2714 from SGX Sensortech, Corcelles-Cormondreche, Switzerland and GGS7530 from UST Umweltsensortechnik, Geschwenda, Germany). We used a homemade diffuser system to control and generate different concentrations of NO_2_ in pure air (Figure 1). Two mass flow controllers were used to monitor the flow of the pure air and nitrogen dioxide cylinders. The total flowrate was set to 400 mL/min and we could generate different concentrations by varying the percentage of nitrogen dioxide flow over the total flow. The nitrogen dioxide cylinder had a dilution of 10 ppm (parts per million). Therefore, to generate 250 ppb, we had to dilute 10 mL/min of nitrogen dioxide in 390 mL/min of pure air. Nitrogen dioxide and pure air were first introduced in a gas-mixing chamber to ensure the homogeneity of the gas before crossing to the sensors cell which contained two identical electronic noses. We collected 508 measurements. Each measurement took 300 s to reach the steady state response of all sensors. The 508 measurements were distributed between different concentrations of NO_2_ ranging from 25 ppb to 250 ppb, with a step of 25 ppb in order to simulate the real pollution rates found near highways. The collected data were organized in two matrices: $X1_{N}^{D}$ for unit 1 and $X2_{N}^{D}$ for unit 2. Their corresponding known concentration was grouped in $Y_{N}$:

$$X1_{N}^{D}=\left[\begin{array}{ccc} \begin{array}{c} x1_{1}^{1} \end{array} & \ldots & x1_{1}^{D}\\ \vdots & \ddots & \vdots \\ x1_{N}^{1} & \ldots & x1_{N}^{D} \end{array}\right]\;X2_{N}^{D}=\left[\begin{array}{ccc} \begin{array}{c} x2_{1}^{1} \end{array} & \ldots & x2_{1}^{D}\\ \vdots & \ddots & \vdots \\ x2_{N}^{1} & \ldots & x2_{N}^{D} \end{array}\right]\;Y_{N}=\left[\begin{array}{c} y_{1}\\ \vdots \\ y_{N} \end{array}\right]\;$$

where N = 508 represents number of samples and D = 900 is the dimension of samples composed from 300 data points for each of the three sensors.

## 3. Calibration Transfer

Before introducing our proposal method, let us first present the DS method. We considered $X1_{N}^{D}$ and $X2_{N}^{D}$ to be the data matrices of the master unit and the slave unit, respectively, where N is the number of samples and D is the dimension of a sample. To match between these two units, we needed to select a subset of samples from both of them. $S1_{m}^{D}$ and $S2_{m}^{D}$ were the response matrices containing m samples selected respectively from each of $X1_{N}^{D}$ and $X2_{N}^{D}$. The classical DS was based on using the transformation matrix *F* given by:

(1) $$\;F=S2^{+}\times S1\;$$

where $S2^{+}$ is the generalized or pseudo-inverse of S2.

Data from the slave unit can be standardized as:

(2) $$\;X2_{stand}=X2\times F$$

Once the data from the slave unit was standardized, concentrations could be predicted using the calibration model of the master unit. Direct standardization was based on two essential elements:

1. An algorithm that can select the standardization samples in a way to be as representative as possible of the entire dataset.
2. A technique that can match the two units with a fewer number of standardization samples.

In our proposal method, we used kernel SPXY to select the standardization samples and we replaced the matrix transformation by applying SVM regression between S2 and S1.

### 3.1. Kernel SPXY

All data selection algorithms used the same principle and were based on calculating the distance between samples. The algorithms started by taking the pair (p,q) of samples that had the largest distance d(p,q). To select a new sample, the distances of all the remaining samples with respect to all samples already selected were calculated. For each sample, these algorithms kept their minimum distance regarding other selected samples. The sample to be selected should then have the maximum distance. These algorithms stopped when they achieved the desired number of samples to be selected. The only difference between these algorithms was in their manner of calculating the distance between samples. The Kennard-Stone algorithm uses the Euclidian distance and is given by:

(3) $$\;d_{x}\left(p,q\right)=\sqrt{\overset{D}{\underset{i=1}{\sum }}{\left(x_{p}\left(i\right)-x_{q}\left(i\right)\right)}^{2}}\;$$

where p,q are samples and D is the dimension.

SPXY algorithms take in consideration not only data response but also target values (concentrations) to calculate the distance between two samples:

(4) $$\;d_{xy}\left(p,q\right)=\frac{d_{x}\left(p,q\right)}{max_{\left(p,q\right)\epsilon \left[1,N\right]}d_{x}\left(p,q\right)}\;+\frac{d_{y}\left(p,q\right)}{max_{\left(p,q\right)\epsilon \left[1,N\right]}d_{y}\left(p,q\right)}$$

(5) $$\;d_{xy}\left(p,q\right)=\frac{\sqrt{\sum_{i=1}^{D}{\left(x_{p}\left(i\right)-x_{q}\left(i\right)\right)}^{2}}}{max_{\left(p,q\right)\epsilon \left[1,N\right]}d_{x}\left(p,q\right)}\;+\frac{\sqrt{{\left(y_{p}-y_{q}\right)}^{2}}}{max_{\left(p,q\right)\epsilon \left[1,N\right]}d_{y}\left(p,q\right)}$$

The kernel SPXY algorithm is a modified version of the SPXY algorithm in which the Euclidian distance of the SPXY algorithm is replace by a kernel distance (kd) given by:

(6) $$\;kd_{xy}\left(p,q\right)=\frac{kd_{x}\left(p,q\right)}{max_{\left(p,q\right)\epsilon \left[1,N\right]}kd_{x}\left(p,q\right)}\;+\frac{kd_{y}\left(p,q\right)}{max_{\left(p,q\right)\epsilon \left[1,N\right]}kd_{y}\left(p,q\right)}$$

where

(7) $$\;kd_{x}\left(p,q\right)=\sqrt{\overset{D}{\underset{i=1}{\sum }}K\left(x_{p}\left(i\right),x_{p}\left(i\right)\right)+\overset{D}{\underset{i=1}{\sum }}K\left(x_{q}\left(i\right),x_{q}\left(i\right)\right)-2\overset{D}{\underset{i=1}{\sum }}K\left(x_{p}\left(i\right),x_{q}\left(i\right)\right)}$$

and

(8) $$\;kd_{y}\left(p,q\right)=\sqrt{K\left(y_{p},y_{p}\right)+K\left(y_{q},y_{q}\right)-2K\left(y_{p},y_{q}\right)}$$

For further details about kernel SPXY, the reader can refer to Gani et al. [21] in which the authors concluded that kernel SPXY algorithm performs better than the SPXY and Kennard-Stone algorithms.

### 3.2. Support Vector Machine Regression

Support vector machine regression is a machine learning method widely used for building calibration models [22]. Its structure guarantees a good generalization and accuracy with a limited number of learning samples [23]. Support vector machine establishes a linear regression function between dataset x and the target values y as follows:

(9) $$\;f\left(x_{i},w\right)=w.x_{i}+b\;$$

where *w* is the regression coefficients vector and b is the bias term.

In order to calculate the regression coefficients, SVM regression attempts to minimize the loss function as defined by:

(10) $$\;{\left|y_{i}-f\left(x_{i},w\right)\right|}_{\text{є}}=\{\begin{array}{c} 0\\ \left|y_{i}-f\left(x_{i},w\right)\right|-\varepsilon \; \end{array}\begin{array}{c} \;\mathrm{if}\;\left|y_{i}-f\left(x_{i},w\right)\right|\leq \varepsilon \\ \mathrm{otherwise} \end{array}\;$$

where ε is the maximum difference between the predicted value and the target value that can be neglected.

In order to simultaneously minimize the empirical risk and model complexity, support vector machine regression was formulated as an optimization problem with constraints:

(11) $$\mathrm{Minimizing}:\frac{1}{2}{||w||}^{2}+C\overset{n}{\underset{i=1}{\sum }}\left(\xi_{i}+\xi_{i}^{*}\right)\;$$

(12) $$\;\mathrm{Subject}\;\mathrm{to}\;\{\begin{array}{c} y_{i}-f\left(x_{i},w\right)-b\leq \varepsilon +\xi_{i}^{*}\\ f\left(x_{i},w\right)+b-y_{i}\leq \varepsilon +\xi_{i}\\ \xi_{i}^{*},\xi_{i}\geq 0 \end{array}\;$$

where C is a constant regularization parameter which determines the tradeoff between the flatness of f(x) and the empirical risk. $\xi_{i}^{*},\xi_{i}$ are slack variables which measure deviations larger than ε.

Using Lagrange multipliers and the Karush-Kuhn-Tucker conditions, we can get the following solution:

(13) $$\;f\left(x\right)=\overset{n}{\underset{i=1}{\sum }}\left(\alpha_{i}-\alpha_{i}^{*}\right)K\left(x_{i},x\right)+b\;$$

where $\alpha_{i}$ and $\alpha_{i}^{*}$ are the Lagrange multipliers and $K\left(x_{i},x\right)$ constitutes the kernel function. The kernel function used in this work was the Gaussian kernel defined as follows:

(14) $$\;k\left(x_{i},x_{j}\right)=exp\left(\frac{-{||x_{i},x_{j}||}^{2}}{2\delta^{2}}\right)\;$$

where δ is the standard deviation of the kernel.

In any machine learning, the results depend on a good choice of their hyperparameters. For this work, we used the generalized pattern search (GPS) [24] to optimize SVM regression hyperparameters by minimizing the cross validation prediction error. The parameters considered were:

- Gaussian kernel with δ = 30
- The constraint C = 2000
- Epsilon-insensitive band ε = 0.0001

**Figure 2:**
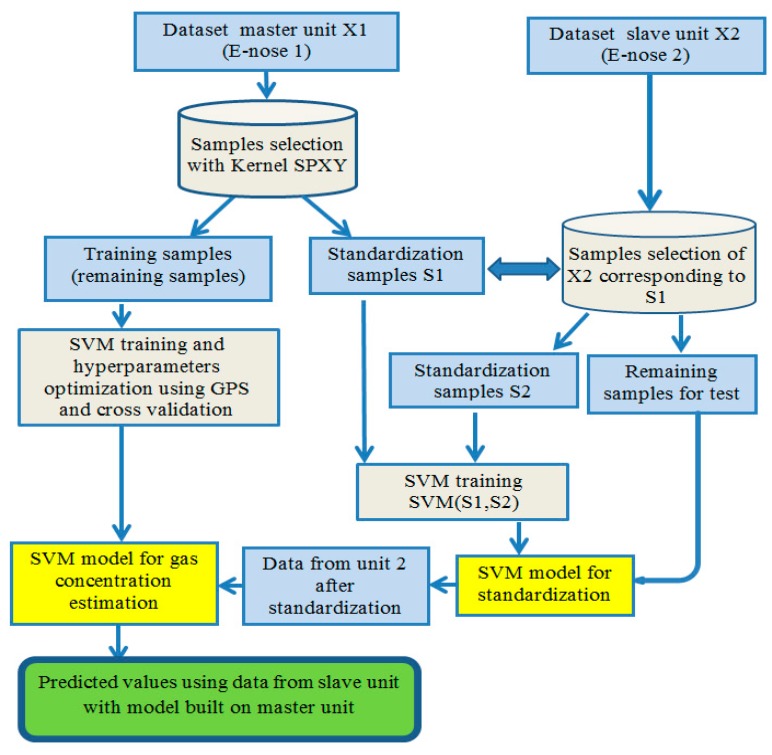
Illustration of the calibration transfer procedure between two identical electronic noses using support vector machine regression (SVM).

Figure 2 summarizes our procedure for calibration transfer. We used kernel SPXY to select the desired number of standardization samples from the dataset of the master unit (E-nose 1). The remaining data were used to build a model calibration using SVM regression. Support vector machine regression hyperparameters were optimized using generalized pattern search (GPS). Next, from the slave unit (E-nose 2), we selected the analogue samples of the selected samples from the master unit. SVM regression was used again to create another model used to standardize data from the slave unit. Finally, we could predict concentration using the model calibration built on the master unit with data from the slave unit.

## 4. Results and Discussion

**Figure 3:**
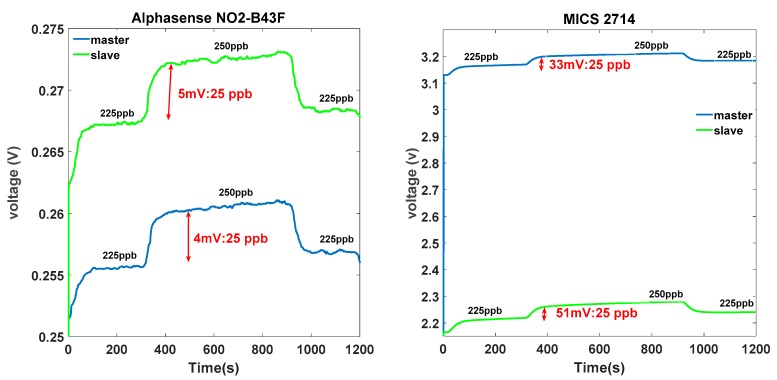
Acquired responses signals from identical sensors under the same conditions. ppb = parts per billion.

Signal responses acquired from two identical electronic noses exposed to the same gas concentration under the same conditions were different in terms of sensitivity and baseline. Figure 3 presents the signal acquired from NO2-B43F Alphasense sensors and MICS-2714 sensors of the master unit and the slave unit. Even if the master and the slave units have undergone the same history of use, the signals collected from the same sensor type differed in terms of baseline and sensitivity. For example, concerning the baseline between the two electrochemical sensors, we observed a shift of about 12 mV, which can correspond to 75 ppb of NO_2_. Additionally, for the two MOX sensors, the difference in baseline was very important. This considerable shift in MOX sensors may have been due to the large resistance margin of the initial resistor provided by the constructor (the initial resistor of GGS 7530 was 50 kΩ ± 35 kΩ). Concerning the sensitivity, we used a numerical indicator in Figure 3 to indicate the variation because of the very low signal scale. For example, in the case of the MICS-2714 sensors, the sensor sensitivities were 33 mV and 51 mV, respectively, for 25 ppb of NO_2_. This slight difference in terms of sensitivity could significantly deviate the NO_2_ estimation with regard to the very low concentration range (0–250 ppb). As a consequence, any model built on E-nose 1 could not be useful for E-nose 2.

**Figure 4:**
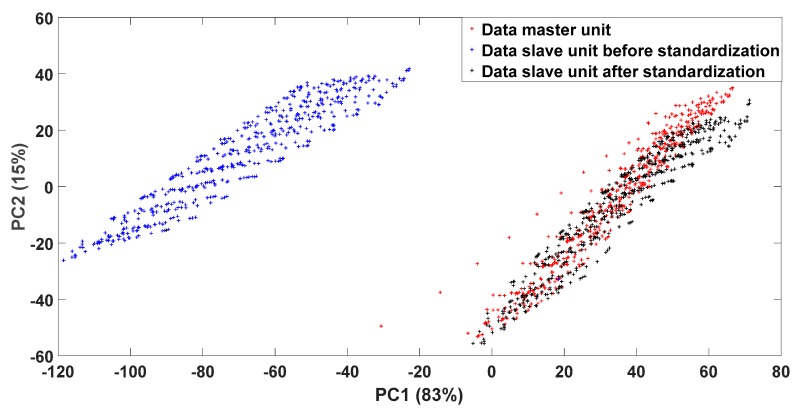
Principal component analysis (PCA) projection of the data captured with unit 1 and signals from unit 2 before and after standardization.

To show the ability of the proposed method to create a match between the sensor responses of a master unit and a slave unit, we used principal component analysis (PCA) projection. We plotted the first two PCA components of all the data obtained from the master and the slave units before and after the standardization. Figure 4 shows that the data from each unit are gathered in different regions. By the use of only 10 samples for standardization, the data from the slave unit were shifted to the region of the master unit and they became more alike.

**Figure 5:**
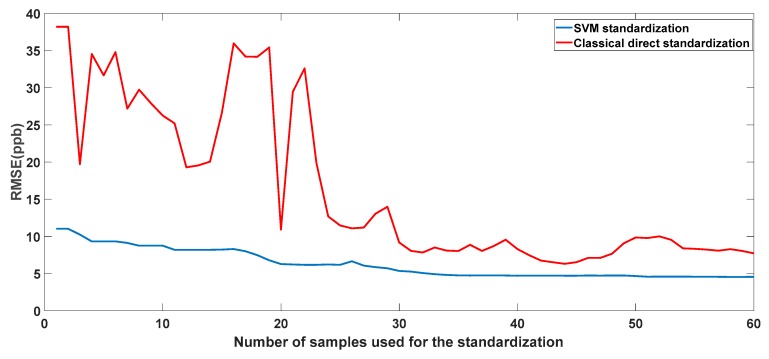
Evolution of the root mean square error (RMSE) prediction as a function of standardization sample number in two cases: classical direct standardization and SVM standardization.

Our method was compared with the classical Direct Standardization. First, we built a calibration model on the master unit using SVM regression. Then, we used kernel SPXY to select a number of standardization samples. After standardization of the slave unit data, we used the calibration model built on the master unit to predict NO_2_ concentrations. We repeated this procedure 60 times by adding in one sample for standardization each time. We determined the root mean square error prediction using 10-fold cross validation. In Figure 5, we show that our method outperformed the classical DS. This method needed 10 samples to reach a prediction accuracy around 8.7 ppb while the classical DS method needed a least 30 samples to reach a stable and acceptable accuracy.

**Figure 6:**
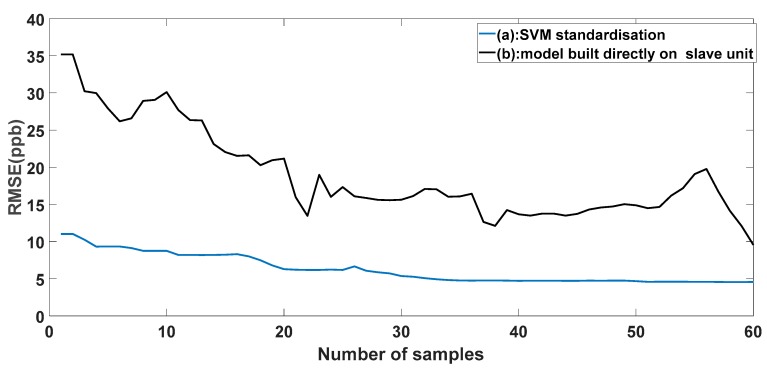
(**a**) Evolution of root mean square error as a function of the standardization sample number by using the master calibration model. (**b**) Evolution of root mean square error as a function of the sample number used for building a new model directly on the slave unit.

The aim of any calibration transfer method is to preserve time and prevent the high cost of a new calibration. In fact, if the standardization procedure needed as many samples as the master unit to build a new model for the slave unit, it is not useful for making the calibration transfer, and it would be better to calibrate each unit individually. So, we tested whether the number of samples used for standardization was enough to build a new calibration model directly on the slave unit. We used the same selected standardization samples to directly build a new calibration model on the slave unit. We started the operation from one sample to 60 samples by adding in one sample each time. In each time, we calculated the root mean square error prediction of 10-fold cross validation in the two cases: in the case of using the samples selected for standardization and in the case of using these samples to build a new calibration model on the slave unit. In Figure 6, we plotted the evolution of the error prediction along with the number of samples. The figure shows that building a new calibration model needed a least 60 samples to obtain an acceptable prediction accuracy.

**Figure 7:**
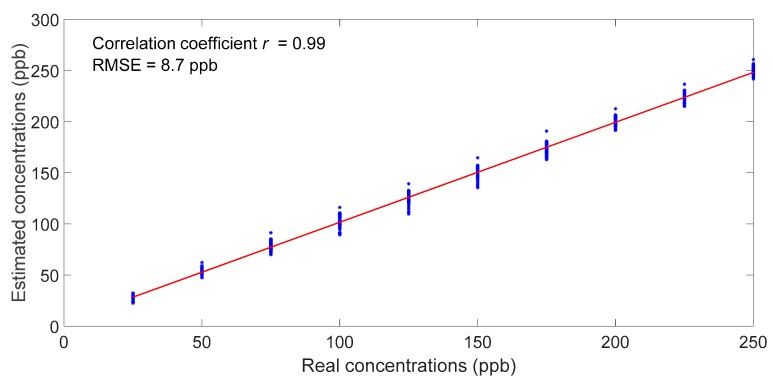
Performance of SVM standardization using 10 samples, illustrated by the predicted concentrations over the real concentrations.

Figure 7 shows the predicted concentrations as a function of the real concentrations. The predicted values were obtained using a calibration model built on the master unit and the standardized data from the slave unit with 10 samples used for standardization. The estimated values and the real values were almost superposed. This was numerically confirmed by calculating the root mean square error prediction of 10-fold cross validation, which was equal to 8.7 ppb.

## 5. Conclusions

Air pollution monitoring systems based on low cost sensors is a promising instrument that can complement the actual air pollution monitoring network. In this work, we built two identical electronic noses to study the performance of the calibration transfer. First, we showed that both electrochemical and metal oxide sensors can detect a low range of concentrations that corresponded to the real concentrations near highways. We saw that two identical electronic noses under the same conditions provided different signals in terms of the baseline as well as the sensitivity. The goal of our study was to develop a new approach to make a calibration transfer with a minimum number of samples used for matching between units. To perform the calibration transfer, two essential considerations should be taken into account: the selection of a subset of samples that should be as representative as possible for the entire dataset, and finding a mathematical method that needs as few as possible samples to make the relation between data units. So, we utilized kernel SPXY for data selection and SVM regression for data standardization. Then, we demonstrated that the calibration model of the master unit could be used with success on the slave unit after the standardization. The results showed that this method provided better performances than classical direct standardization. Our next challenge is to test the proposed method in real conditions by lodging the two electronic noses in government monitoring stations and adopting the analyzer as a reference instrument.
